# Integrin αvβ6-specific therapy for pancreatic cancer developed from foot-and-mouth-disease virus

**DOI:** 10.7150/thno.38702

**Published:** 2020-02-12

**Authors:** Kate M. Moore, Ami Desai, Bea de Luxán Delgado, Sara Maria David Trabulo, Claire Reader, Nicholas F. Brown, Elizabeth R. Murray, Adam Brentnall, Philip Howard, Luke Masterson, Francesca Zammarchi, John A. Hartley, Patrick H. van Berkel, John F. Marshall

**Affiliations:** 1 Barts Cancer Institute, Queen Mary University of London, John Vane Science Centre, Charterhouse Square, London EC1M 6BQ, UK.; 2 Cancer Research UK Centre for Epidemiology, Mathematics and Statistics, Wolfson Institute of Preventative Medicine, Queen Mary University of London, Charterhouse Square, London EC1M 6BQ, UK.; 3 Spirogen, QMB Innovation Centre, 42 New Road, London E1 2AX, UK.; 4 ADC Therapeutics (UK) Ltd, QMB Innovation Centre, 42 New Road, London E1 2AX, UK.; 5 Cancer Research UK Drug-DNA Interactions Research Group, University College London Cancer Institute, 72 Huntley Street, London WC1E 6BT, U.K.

**Keywords:** integrin, αvβ6, PDAC, peptide-drug conjugate

## Abstract

**Goals of investigation**: The 5-year survival rate for pancreatic ductal adenocarcinoma (PDAC) has remained at <5% for decades because no effective therapies have been identified. Integrin αvβ6 is overexpressed in most PDAC and represents a promising therapeutic target. Thus, we attempted to develop an αvβ6-specific peptide-drug conjugate (PDC) for therapy of PDAC.

**Methodology**: We conjugated the DNA-binding pyrrolobenzodiazepine (PBD)-based payload SG3249 (tesirine) to an αvβ6-specific 20mer peptide from the VP1 coat protein of foot-and-mouth-disease virus (FMDV) (forming conjugate SG3299) or to a non-targeting peptide (forming conjugate SG3511). PDCs were tested for specificity and toxicity on αvβ6-negative versus-positive PDAC cells, patient-derived cell lines from tumor xenografts, and on two different *in vivo* models of PDAC. Immunohistochemical analyses were performed to establish therapeutic mechanism.

**Results**: The αvβ6-targeted PDC SG3299 was significantly more toxic (up to 78-fold) for αvβ6-expressing versus αvβ6-negative PDAC cell lines* in vitro*, and achieved significantly higher toxicity at equal dose than the non-targeted PDC SG3511 (up to 15-fold better). Moreover, SG3299 eliminated established (100mm^3^) Capan-1 PDAC human xenografts, extending the lifespan of mice significantly (P=0.005). Immunohistochemistry revealed SG3299 induced DNA damage and apoptosis (increased γH2AX and cleaved caspase 3, respectively) associated with significant reductions in proliferation (Ki67), β6 expression and PDAC tumour growth.

**Conclusions**: The FMDV-peptide drug conjugate SG3299 showed αvβ6-selectivity *in vitro* and *in vivo* and can specifically eliminate αvβ6-positive cancers, providing a promising new molecular- specific therapy for pancreatic cancer.

## Introduction

Pancreatic ductal adenocarcinoma (PDAC) is the fourth most common cause of cancer-related deaths 1 with a 5-year survival rate of less than 5% 2, predominantly due to clinical signs of disease occurring late in disease progression, often after metastasis has occurred. Current treatment options are extremely limited, with surgical resection and chemotherapy only effective in cases that are detected early and when disease is restricted to the pancreas. Combination chemotherapy strategies such as FOLFIRINOX (fluorouracil, leucovorin, irinotecan and oxaliplatin) and gemcitabine with nab-paclitaxel have significantly improved survival compared to gemcitabine, the previous standard of care for PDAC 3, 4. However, these improvements amount to less than five months additional survival for patients who have metastatic disease, who are the majority of patients. The incidence of pancreatic cancer is increasing and is predicted to be the second highest cause of cancer-death in the USA by 2030 5. Thus, there remains an urgent unmet clinical need to develop effective targeted therapies.

Integrins are heterodimeric transmembrane receptors which mediate many biological functions including adhesion, migration, invasion, growth, survival and differentiation of cells 6. Deregulation of integrin expression or signaling is associated with cancer development and metastasis. Integrin αvβ6 is overexpressed in over a third of carcinomas 7 in a variety of cancers including breast 8, cervical 9, colon 10, and non-small cell lung cancer 11, where its expression correlates with poor overall survival. We 12, and others 13,14, have reported that integrin αvβ6 is also overexpressed in most PDAC, whereas it is low or absent in normal pancreas, making αvβ6 a biomarker for disease but also a potential therapeutic target. In fact, we have shown antibody blockade of αvβ6 in human pancreas xenografts and mouse syngeneic tumours significantly slowed the growth of established tumours *in vivo* and increased survival 12. In this study, we sought to deliver a cytotoxic drug specifically to kill αvβ6-expressing pancreatic cancer cells* in vivo*.

To achieve cell-specific delivery of drugs requires a molecular-specific vector conjugated to a cytotoxic warhead. In earlier studies 7,15,16 we discovered that a 20 amino-acid sequence NAVPNLRGDLQVLAQKVART (termed A20FMDV2) from the VP1 coat protein of the O_1_ BFS serotype of foot-and-mouth-disease-virus (FMDV), bound specifically and with high affinity (<1 nM) only to integrin αvβ6, resulting in αvβ6-dependent internalisation. Therefore we conjugated A20FMDV2, modified to include an N-terminal biotin and a C-terminal cysteine, to a cytotoxic payload, SG3249 (tesirine) 17. Tesirine is comprised of a cathepsin B-cleavable valine-alanine linker conjugated to a pyrrolobenzodiazepine (PBD) warhead, SG3199. SG3199 is a potent PBD dimer toxin which covalently cross-links DNA in the minor groove, preventing replication and resulting in cell death 18. Tesirine is currently being tested in clinical trials conjugated to monclonal antibodies to CD25 (NCT02432235; NCT02588092), CD19 (NCT02669017; NCT02669264), PSMA (NCT02991911) and DLL3 (NCT01901653) 19.

To our knowledge, no drugs that target αvβ6 in cancer are currently in pre-clinical development. We show here for the first time that by conjugating tesirine to the FMDV-derived peptide A20FMDV2 to create SG3299, we can selectively direct the DNA damaging activity of the PBD dimer to αvβ6-overexpressing tumors and completely eliminate, or prevent development of, established pancreatic cancer xenografts. We suggest SG3299 is a promising new therapy for PDAC and many other carcinomas that upregulate αvβ6.

## Materials & Methods

Additional methods details are described in Supplementary Methods.

### Chemical structures and synthesis

A20FMDV2 and non-targeting (NT) peptides, modified to include N-terminal biotinylation and a C-terminal cysteine which does not affect specificity (see below), were obtained from Peptide Synthetics (>95% purity; Peptide Protein Research Ltd, Hampshire, UK). A20FMDV2 7, 15,16 and SG3199 17 have been characterised previously; SG3249 and SG3199 were prepared as described 17. Drugs were prepared as 1mM stocks in 100% DMSO, aliquoted and frozen at -20ºC. Samples were thawed and diluted directly into PBS; the PBS only no-drug controls had equivalent concentrations of DMSO added. See Supplementary Figure S1 for conjugate structures and Supplementary Methods for conjugation procedures and analytical liquid chromatography/mass spectrometry (LC/MS) conditions for reaction monitoring, HPLC purity determination and manufacture of SG3511 and SG3299.

### Cell culture

A375P-Puro and A375P-β6 cell lines were developed in-house and have been characterized previously 15. All other cell lines were provided by colleagues in Barts Cancer Institute. All cell lines were authenticated by LGC STR profiling (data not shown) and utilised within 6 months of resuscitation. A375Ppuro, A375Pβ6, Panc1 and Colo357 cell lines were grown in DMEM+10%FBS. Capan-1 and Panc0403 cells were cultured in RPMI+10%FBS. PS1 cells were grown in DMEM:HamsF12+10%FBS. PANC354, PANC253 and PANC215 cells derived from 3 human PDX PDAC models were a generous gift from Professor James Eshleman (John Hopkins University, USA) and were cultured as previously described 20.

### Sphere-forming assay

500 cells/well of PANC354, PANC253 and PANC215 cells (hereafter termed 354, 253 & 215) were grown as adherent cells in 6-well plates. After 24h cells were treated with 1 nM, 10 nM, 50 nM, 100 nM and 500 nM PDCs or control containing DMSO. After 3 days, cells were collected and re-seeded at 8 x 10^3^ cells/well of a 24-well plate (n=5-6 wells/treatment). After 10 days the number of spheres were counted. Photographs of spheres after 10 days were taken using an Olympus CKX31 inverted microscope and camera (Olympus Corporation, Japan) at x4 magnification.

### Human tumor xenograft models

All animal experiments followed UK Home Office Guidelines. 8-week old female CD1Nu/Nu-mice (Charles River, UK) were inoculated subcutaneously with 2x10^6^ A375Ppuro, A375Pβ6 or Capan-1 cells in 200 μl of PBS. For PDAC/ stellate xenograft studies 1x10^6^ Panc0403 were co-injected with 2x10^6^ PS1 pancreatic stellate cells in PBS:Matrigel (2:1 ratio) into 8-week old female CD1Nu/Nu-mice. Mice were randomized into treatment groups once the starting tumor volume reached ~100 mm^3^. Mice received either tri-weekly intraperitoneal injections (10 μg/kg in 200 µl of PBS) or bi-weekly intraperitoneal injections (20 or 25 μg/kg in 200 µl of PBS) for 4-5 weeks of NT-peptide control, A20FMDV2, SG3199, SG3511 or SG3299 (n=4-8/treatment). PBS control contained the equivalent amount of DMSO vehicle as peptide treatments. Tumors were measured with calipers bi-weekly in two directions and tumor volume calculated using the formula (width^2^ x length)/2. Animals were weighed at least bi-weekly to calculate accurate dosing and also observed for behavioural changes as a measure of possible toxicity. Tumors, organs and tissues were harvested at the end of each study for immunohistochemical analyses. In addition, some Capan-1 tumors, tissues and organs (n=3/treatment) were also harvested after 11 days (after 3 treatments) when treated with 25 μg/kg bi-weekly.

### Peptide-drug conjugate serum stability assay

8-week old female CD1Nu/Nu-mice were treated with 20 μg/kg SG3299 in 200 µl of PBS by intraperitoneal injection for 2 minutes, 5 minutes, 15 minutes or 60 minutes. Untreated animals served as controls (n=4/condition). At the end of each time point, blood was collected via cardiac puncture under terminal anaesthesia and allowed to coagulate overnight at 4°C. Serum from each sample was then isolated by centrifugation at 1000 g for 20 minutes. The serum concentration of SG3299 was subsequently determined by enzyme-linked immunosorbent assay (ELISA). In brief, peptide-drug conjugate was captured with 1.5 μg/ml immobilised α-PBD antibody (14B3-B7, ADC Therapeutics) synthesised as described 21. Peptide-drug conjugate was then detected by incubation with Streptavidin-HRP (N200, Thermo Fisher) followed by the addition of 3,3'5,5'-Tetramethylbenzidine (TMB) substrate (34028, Thermo Fisher), neutralisation with 1M HCl, and measurement of absorbance at 450 nm minus absorbance at 620 nm. SG3299 concentration was determined by subtraction of absorbance readings from untreated mice and interpolation relative to reconstituted SG3299 standards of known concentration in GraphPad Prism software (Systat Software, San Jose, CA, USA).

### Immunohistochemical analysis

Immunohistochemistry utilized 4 μm, formalin-fixed, paraffin-embedded serial sections of tumors. Tumor sections were stained for molecules of interest including epithelial markers pan-cytokeratin (CK) (M3515, DAKO) and E-cadherin (610181, BD Bioscience), marker of DNA damage γH2AX (AB22551, Abcam), proliferation marker Ki67 (AB92742, Abcam), αvβ6 (mAb 6.2E2, Biogen Idec), apoptotic marker cleaved-caspase 3 (9661, Cell Signaling Technology), endomucin for detection of endothelial cells (SC-53941 V.5C7, Santa Cruz), vimentin (V5255, Sigma) for detection of stromal mesenchymal cells (mostly fibroblasts) and α-sma (M0581 clone 1A4,DAKO) for detection of activated fibroblasts/ stellate cells. The protocol used for αvβ6 integrin was described previously 8. Staining for CK and αvβ6 was perfomed manually, all other staining was performed using the DISCOVERY XT automated IHC research slide staining system (Ventana Medical Systems Inc.).

### Statistical analysis

IC_50_ values for *in vitro* analyses were obtained using GraphPad Prism software (Systat Software, San Jose, CA, USA). For 2 variables, data were analysed using an unpaired two-tailed student t-test and for 3 or more variables data were analysed using one-way ANOVA with Bonferroni's Multiple Comparison Test using Prism GraphPad software.

For tumor xenograft models, individual growth curves were plotted and a linear mixed model 22 was used to test for differences between treatment arms, fitted by maximum likelihood using the nlme package in the statistical software R 3.1.1 23. P values are from Wald tests. All statistical tests were two-sided. Error bars in all experiments represent standard deviation (SD) in *in vitro* studies and standard error of the mean (SEM) in *in vivo* studies.

## Results

### *In vitro* Studies Demonstrate αvβ6-Specific Cytotoxicity of SG3299

To create an αvβ6-specific targeted therapy, we modified our FMDV-derived αvβ6-specific peptide A20FMDV2 by addition of an N-terminal biotin, for antibody detection of the resulting conjugate, and addition of a C-terminal cysteine to permit conjugation to the payload tesirine (SG3249), which contains the drug SG3199 linked to a cathepsin B-cleavable linker. The resulting conjugate created an αvβ6-specific peptide-drug conjugate (PDC) SG3299 (see below). A non-targeting (NT) peptide with a random sequence 7 and no RGD motif was conjugated to tesirine creating SG3511, which was used as a PDC control to SG3299. The sequences and structural diagrams of these peptides and PBD drug are shown in Supplementary Figure S1A. The purity of these peptides was assessed using high performance liquid chromatography-mass spectrometry (Supplementary Figure S1B and supplementary methods).

To confirm that the chemistry required to conjugate the linker-drug moiety to the peptide did not affect integrin specificity, we used flow cytometry with anti-biotin antibodies to measure conjugate binding to an isogenic matched pair of cell lines A375Ppuro and A375Pβ6, the latter engineered to overexpress αvβ6 7. Supplementary Figures S2A & S2B show that SG3299 exhibited dose-dependent binding to A375Pβ6 cells from 0.1 to 100 nM but failed to bind to A375Ppuro cells at even 100 nM. Mean fluorescence intensity (MFI) was used as a measure of binding.

In contrast, the control SG3511 failed to bind to either cell line at any concentration tested (100 nM dose shown). Thus, SG3299 retained specificity for αvβ6.

**Figure 1:**
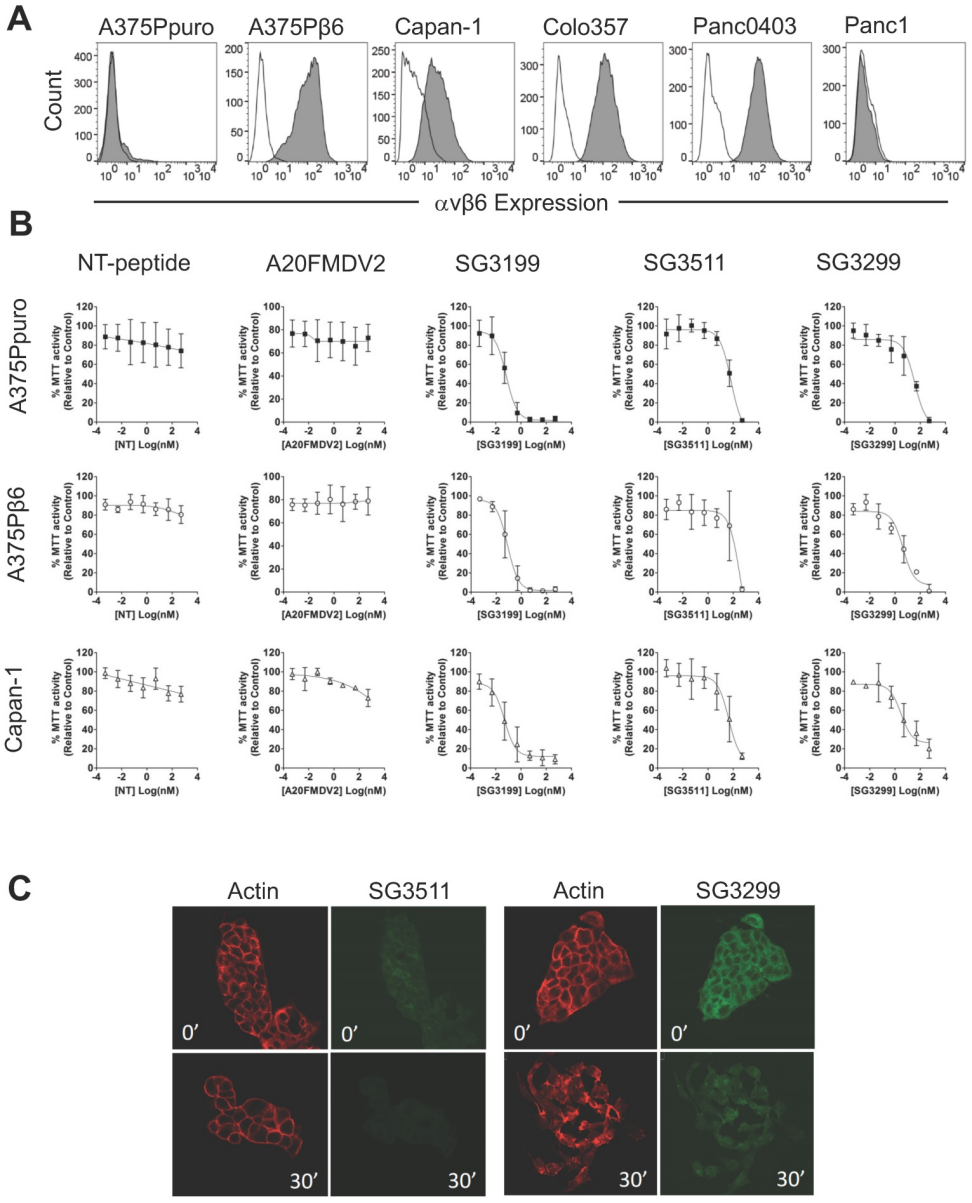
** SG3299 Selectively Kills αvβ6-Expressing Cells. A**, αvβ6 expression of a panel of six cell lines using flow cytometry. Isotype control IgG is unshaded and gray shaded peaks represent αvβ6 expression. **B,** MTT growth inhibition assays were performed in the panel of cell lines in (A). Isogenic matched cell lines A375Ppuro and A375Pβ6 and αvβ6-positive pancreatic cell line Capan-1 are shown as examples. Cells were treated with 0-500nM of non-targeting (NT) peptide, αvβ6-specific peptide A20FMDV2, warhead only SG3199, NT-peptide conjugated to SG3249 (SG3511) and A20FMDV2 conjugated to SG3249 (SG3299) over 72h. All assays were performed in quadruplicate with a minimum of three biological repeats. **C**, Internalisation of SG3511 and SG3299 in Capan-1 cells. Cells were treated with 50nM SG3511 or SG3299 for 30 minutes on ice followed by incubation at 37°C. Indirect immunofluorescence was used to detect the biotin tag and Alexa-488 (green) conjugated secondary antibodies revealed the location of conjugates SG3511 and SG3299. The red fluorescence (Phalloidin-TRITC) shows the actin cytoskeleton at the same time-points.

Before proceeding to toxicity assays, we screened a panel of pancreatic cancer cell lines for endogenous αvβ6 expression and chose three positive (Capan-1, Colo357, Panc0403) and one negative (Panc-1) cell line for study (Figure 1A). Figure 1B shows the dose response curves for the A375Ppuro/A375Pβ6 pair of cell lines when treated with NT-peptide, A20FMDV2, SG3199, SG3511 and SG3299. SG3299 was over 15-fold more potent in A375Pβ6 cells than SG3511 and was 5.7-fold more selective for A375Pβ6 cells than A375Ppuro cells as determined by IC_50_ values (Table 1).

There was no significant difference in IC_50_ values for the non-targeting SG3511 in A375Ppuro or A375Pβ6 cells, showing the non-selective toxicity of the NT-peptide-drug conjugate. Neither NT-peptide nor the modified A20FMDV2 had a significant effect on MTT activity (used as an indirect read out of cell proliferation) (Figure 1B).

To validate the toxic effects of the conjugates in pancreatic cancer cells endogenously expressing αvβ6, we tested the panel of pancreatic cancer cell lines (Figure 1B and Table 1). Panc1 cells were included as an example of an αvβ6-negative pancreatic cancer cell line. Panc1 showed limited toxic response to SG3299 compared with the other cell lines tested, but note, also showed a lower toxic response to the free drug. Capan-1 dose response curves are shown as an example of αvβ6-positive pancreatic cancer cell response to the peptides and PDCs (Figure 1B). SG3299 was between 2.8- and 13.2-fold more selective for αvβ6-positive pancreatic cells lines compared with SG3511, confirming that the anti-proliferative activity of SG3299 is more selective for αvβ6-positive pancreatic cancer cells (Table 1).

### SG3299 is internalised only by αvβ6-Positive cells

The payload SG3199, must covalently bind to DNA to induce cytotoxicity 18. To confirm internalisation of SG3299 was occurring in an αvβ6-dependent manner, and thus delivering toxin SG3199 into the cell, we performed internalisation assays in Capan-1 cells. Figure 1C shows there was very little SG3511 associated with cells at 0' or 30' and that the actin cytoskeleton remained unchanged, suggesting no significant toxicity. In contrast, at 0' there was strong binding and entry into the cytoplasm by SG3299, but no SG3299 in the nuclei. At 30', the cells, which usually grow as tightly bound epithelial islands, had undergone significant morphological change, separated into individual cells with irregularly distributed actin, consistent with significant toxicity. Moreover, the green fluorescence was distributed throughout the whole cell by 30', confirming that SG3299 was throughout the cytoplasm and nuclei. Similar results were observed in other pancreatic cell lines (Supplementary Figure S2C). There was no significant difference in the levels of internalised SG3299 or SG3511 in the αvβ6-negative Panc1 cell line. Together, these data confirm that SG3299 binding and internalisation was αvβ6-specific.

### SG3299 inhibits sphere formation in αvβ6-expressing Patient-derived Xenograft PDAC cells

**Figure 2:**
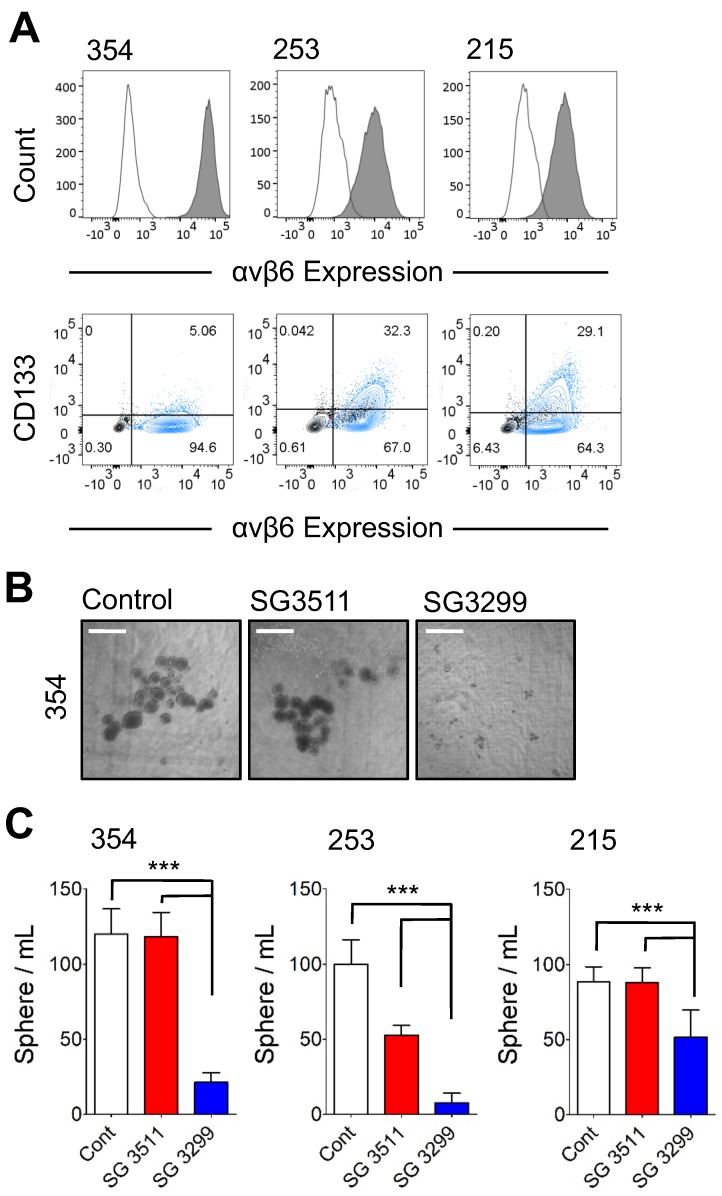
** SG3299 Inhibits Sphere Formation in αvβ6-Expressing Patient-Derived Xenograft PDAC cells. A**, αvβ6 (*upper panel*) and CD133 expression of 354, 253 and 215 PDX PDAC cells using flow cytometry. Isotype control IgG is unshaded and gray shaded peaks represent αvβ6 expression. *Lower panel*, representative co-expression of CD133 and αvβ6 where the majority of CD133+ cells express αvβ6. Isotype control is shown in black and the stained cell population in blue (n=2-5 replicates). **B**, Representative images of 354 cell spheres formed after 10 days culture following 3 days pre-treatment with Control (DMSO), NT-peptide SG3511 or SG3299 (1 nM) (X4 magnification, scale bar=500μum). **C,** Histograms quantifying the sphere formation assay shown in (B) (error bars represent standard deviation, n=5-6 wells/treatment). ***P<0.001 (relative to NT-peptide or SG3511 treatment arm).

Cancer cells with 'stem-like' properties have been linked to tumor initiation, metastasis and development of resistance to therapy 24-26. Formation of spheres *in vitro* that develop from individual cells is reported to enrich for the 'stem' cell phenotype in PDAC cells 20, 25. Hence, to assess the ability of the PDCs to target PDAC cells with 'stem-like' properties, we performed a sphere-forming assay. In preliminary experiments three patient-derived xenografts (PDX) cell populations (PANC354, PANC253 and PANC215; a kind gift from Dr Eshleman, John Hopkins University) were screened for αvβ6 and CD133 expression. Previous studies showed the tumour-initiating population of PDAC cells required CD133 expression 25. The PDX cells were between 4.8%-31.1% positive for CD133 and also showed >90% expression of β6 (Figure 2A), and over 95% of CD133 cells were αvβ6-positive. Next, the 354, 253 and 215 cells were subject to a sphere-forming assay. Prior to sphere formation, cells were treated for 3 days with the PDCs. Cells were then collected and re-seeded to form spheres. Representative images of 354 spheres after 10 days (from start of treatment) are shown in Figure 2B and quantified in Figure 2C (1 nM data shown as higher doses showed non-selective toxicity). Compared with control-treated spheres, SG3299 significantly reduced the number of spheres formed in all three cell models by 72±27% (P<0.001). The non-targeting SG3511 also exhibited toxicity in 253 spheroids but SG3299 was still significantly more toxic than SG3511 (P<0.001).

### SG3299 selectively kills αvβ6-expressing pancreatic cancers *in vivo*

**Figure 3:**
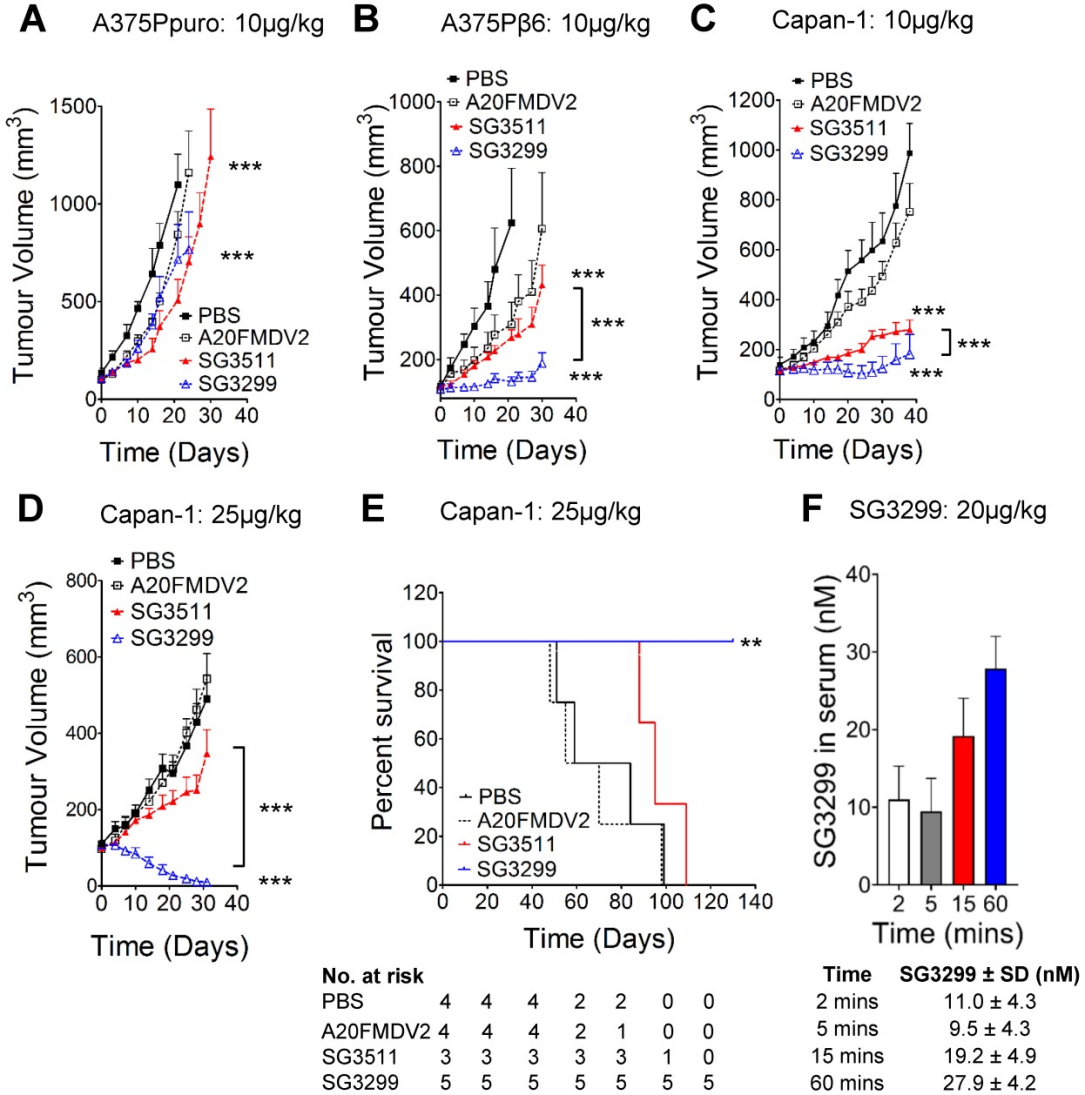
** SG3299 Selectively Kills αvβ6-Expressing Cancers *In Vivo*. A,** Athymic CD1Nu/Nu female mice bearing human A375Ppuro **or B,** A375Pβ6 xenograft tumours were treated with PBS (black square), A20FMDV2 (unfilled square), SG3511 (red filled triangle) or SG3299 (blue unfilled triangle) (10μg/kg; i.p administration) thrice weekly for 4 consecutive weeks (start of treatment day 0). **C,** Mice bearing human Capan-1 tumours were treated as in (A&B) for 5 weeks or **D**, with 25 μg/kg bi-weekly for 4 weeks. Data are presented as mean tumor volume (error bars represent SEM, n=6-8 mice/group). Treatment commenced when tumors reached 100 mm^3^. **E,** Kaplan Meier survival plot of mice from D (excluding mice harvested for immunohistochemical analyses). *P=0.05, **P=0.01, ***P<0.001 (relative to NT-peptide or indicated treatment arm). **F,** Mice were treated with an acute dose of 20 μg/kg SG3299 administered by i.p. injection for 2, 5, 15 or 60 minutes. The concentration of SG3299 in the blood at each time point was determined by ELISA. Data are presented as mean ± SD, n=2-4 mice/group.

We confirmed the specificity of SG3299 to target and deliver the warhead SG3199 to αvβ6-positive tumor cells *in vivo* by performing xenograft studies using cell lines A375Ppuro and A375Pβ6 (Figures 3A & B) which are genetically identical apart from the expression of the β6 integrin subunit. Treatment commenced when tumors were ~100 mm^3^ in all xenograft models. When compared with vehicle (PBS) treated mice, similar levels of tumor growth inhibition were observed in αvβ6-negative A375Ppuro xenografts treated for 4 weeks with 10 μg/kg tri-weekly intra-peritoneal (i.p) injections of non-targeting SG3511 versus αvβ6-specific SG3299. Tumors treated with SG3511 and SG3299 exhibited significant reductions in size (P<0.0001, 53.9±23.7% and 34.8±4.6% after 21 days respectively) but there was no significant difference in the effect of either treatment (P=0.24, after 30 days). These data suggest that SG3199 was delivered into αvβ6-negative cells in a non-selective manner by both conjugates, possibly due to an inherent lipophilicity of SG3199 that acted independently of peptide specificity. In contrast, both SG3299 and SG3511 reduced A375Pβ6 tumor growth compared with PBS treatment (79±7% and 56.9±16.2% respectively, P<0.0001) and SG3299 reduced growth by 2.3-fold more than SG3511 (P<0.0001) (Figure 3B). These data suggest that the specificity of SG3299 for αvβ6 increases the delivery of the cytotoxic warhead to αvβ6-positive tumor cells. A20FMDV2 did not have a significant effect on tumor growth in either A375Ppuro or A375Pβ6 xenograft model (P=0.24).

We then assessed the efficacy of the PDCs using the Capan-1 PDAC xenograft model that endogenously expresses αvβ6. Capan-1 xenografts responded in a similar manner to A375Pβ6 xenografts upon 10 μg/kg tri-weekly treatment, with significant growth inhibition with SG3511 and SG3299 (P<0.0001) (Figure 3C). Again, SG3299 inhibited tumor growth significantly more than SG3511 (P<0.0001).

To investigate whether a higher treatment dose would be even more efficacious in this PDAC model, we repeated the Capan-1 xenograft study with an increased dosage of 25μg/kg but only bi-weekly for 4 weeks (Figure 3D). This dosage was tolerated by mice in preliminary studies (data not shown). Again, SG3299 significantly reduced Capan-1 tumor growth achieving 97.7±2% (P<0.0001) and 96.1±3.4% (P<0.0001) reductions compared to PBS and SG3511, respectively. A20FMDV2 again had no significant effect on tumor growth. The higher dosage of SG3299 eliminated tumors in 4 out of 5 mice and the one remaining tumor was less than 25mm^3^ after 30 days. Several mice receiving this dosing regimen were monitored for survival analysis (Figure 3E). SG3299 treatment conferred a significant survival advantage compared to all other treatments (P<0.043), where all mice were alive after 130 days (4/5 were cured) compared with median survival of 71.5, 62.5 and 95 days for PBS, A20FMDV2 and SG3511-treated mice respectively. Thus, SG3299 significantly prolongs survival of mice bearing Capan-1 xenografts. P<0.0001).

To confirm that intraperitoneal injection of SG3299 could deliver effective levels of PDC to the blood and thus elicit a therapeutic effect on tumour xenografts *in vivo*, we developed an ELISA to detect the serum concentration of SG3299. Capture of the PDC via the C-terminal PBD and subsequent detection via the N-terminal biotin tag allowed us to detect the concentration of intact SG3299 molecules with a sensitivity as low as 0.5nM (Supplementary Figure S3A and S3B). Mice were injected with an acute dose of 20 μg/kg SG3299 and serum samples isolated between 2 and 60 minutes. The concentration of SG3299 in the serum increased over 60 minutes of treatment up to approximately 28nM, well above the effective dose of SG3299 against αvβ6-positive PDAC cells we determined* in vitro* (Table 1). No SG3299 was detected in the urine in the same time frame (data not shown). Thus, intact SG3299 is delivered effectively to the blood and maintained for at least 60 minutes after treatment, indicating that therapeutically active SG3299 is bioavailable following i.p. injection.

### SG3299 treatment reduced proliferation and induced DNA damage and apoptosis in pancreatic tumors

To investigate the molecular effects of PDC therapy on Capan-1 tumors at a cellular level, we harvested Capan-1 xenografts (n=3/treatment) from the xenograft study shown after 3 peptide-drug treatments (tumors for immunochemistry were harvested on day 11, Figure 3D). Immunohistochemistry was performed for a panel of markers to detect DNA damage (γH2AX), residual epithelial cells (cytokeratin (CK) and E-cadherin), proliferation (Ki67), αvβ6, apoptosis (cleaved-caspase3), and endothelial cells as a measure of blood vasculature (endomucin), stromal fibroblasts (vimentin) and activated fibroblasts (α-sma). Tumors were harvested at this time point to provide enough tissue for analysis. The growth curves for the harvested tumors are shown in Supplementary Figure S4A.

No adverse effects were observed with either PDC treatment, as shown by stable mouse weight (Supplementary Figure S4B), normal animal behaviour and by the absence of gross histological change in lung, intestine and stomach (Supplementary Figure S4C-E), three tissues we previously have reported that express endogenous αvβ6 in mice 7.

**Figure 4:**
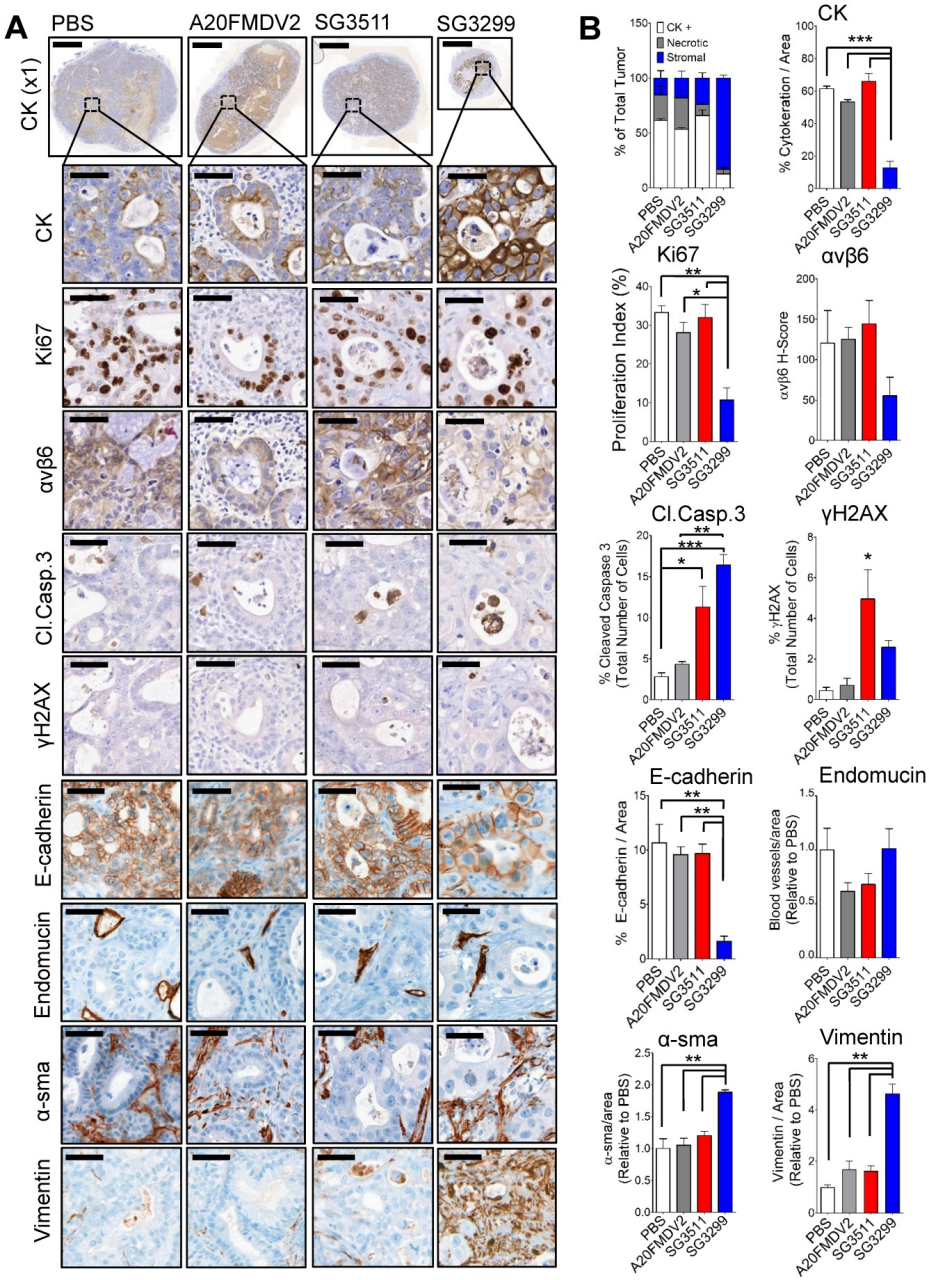
** SG3299 Treatment Reduces Pancreatic Tumor Growth by inducing DNA Damage and Apoptosis. A,** Micrographs of Capan-1 tumor xenografts from mice treated with NT-peptide, A20FMDV2, SG3511 or SG3299 (25 μg/kg; i.p administration) bi-weekly for 10 days (3 treatments) (n=3/treatment tumors harvested after 10 days treatment from Figure 3D). Tumors were harvested, fixed, paraffin-embedded and sections subject to immunohistochemical staining for the indicated molecules of interest including pan-cytokeratin (CK, epithelial marker), Ki67 (proliferation), αvβ6, cleaved-caspase 3 (Cl.caspase 3; apoptosis), E-cadherin (epithelial marker), blood vasculature (endomucin), activated fibroblasts/ stellate cells (α-sma and vimentin), and γH2AX (DNA damage/adduct formation). Representative images are shown of the 3 tumors harvested for each treatment. Magnification bar=2000 μM for x1 magnification images, magnified images (x50) are of indicated region of interest (tumor cells), with a scale bar of 50 μM. **B,** Bar graph of tissue composition of xenografts (% CK+ cells=white bar, % necrotic area in gray and % stroma is in blue) and histograms of marker expression shown in (A). Staining assessed & scored by 2 individuals or using VisioPharm computational analysis software (n=3 individual tumors, error bars represent SEM). *P=0.05, **P=0.01, ***P<0.001 (relative to PBS or indicated treatment).

Representative photomicrographs of immunohistochemical staining are shown (Figure 4A) and were quantified using VisioPharm image analysis software (Figure 4B). There was a significant reduction in proliferation (as determined by reduced Ki67 staining) with SG3299 treatment compared with control (67.5±15%; P<0.01) and compared with SG3511 (66.2±15.7%; P<0.01). This was associated with a marked increase in DNA damage (as measured by 5.9-fold increase in γH2AX) and a reduction in αvβ6 expression of 2.2-fold (54±32.9%) caused by SG3299 treatment compared with PBS (control) treated tumors (Figure 4B).

Compared with PBS (control) treatment, SG3299 was the only treatment to dramatically change tumor composition. For example, compared with PBS (control) treatment there was a significant reduction in CK-positive (79.2±11.2%; P<0.001) and E-cadherin-positive (85±8% (P<0.01) epithelial cells. These observations correlated with a marked increase in DNA damage (γH2AX) and a significant increase in apoptosis as measured by cleaved-caspase 3 (5.8-fold increase from PBS, P<0.001), significantly increased vimentin (4.6-fold compared with PBS, P<0.01) and significantly increased α-sma expression (1.9-fold increase from PBS, P<0.01) (Figure 4B). Thus SG3299-treated Capan-1 tumors became mostly stromal fibroblasts after just 3 treatments with the peptide-drug SG3299.

Non-targeting SG3511 also produced a significant increase in apoptosis and DNA damage (P<0.05) again, probably as a result of the inherent lipophilicity of tesirine, but there was no significant change in tumor cell number, as measured by tumour cell area, CK-expression and confirmed with E-cadherin expression. A20FMDV2 did not elicit any significant tumor changes compared with PBS control treatment as determined by this panel of markers.

### SG3299 selectively kills αvβ6-expressing pancreatic cancers grown with stellate cells *in vivo*

**Figure 5:**
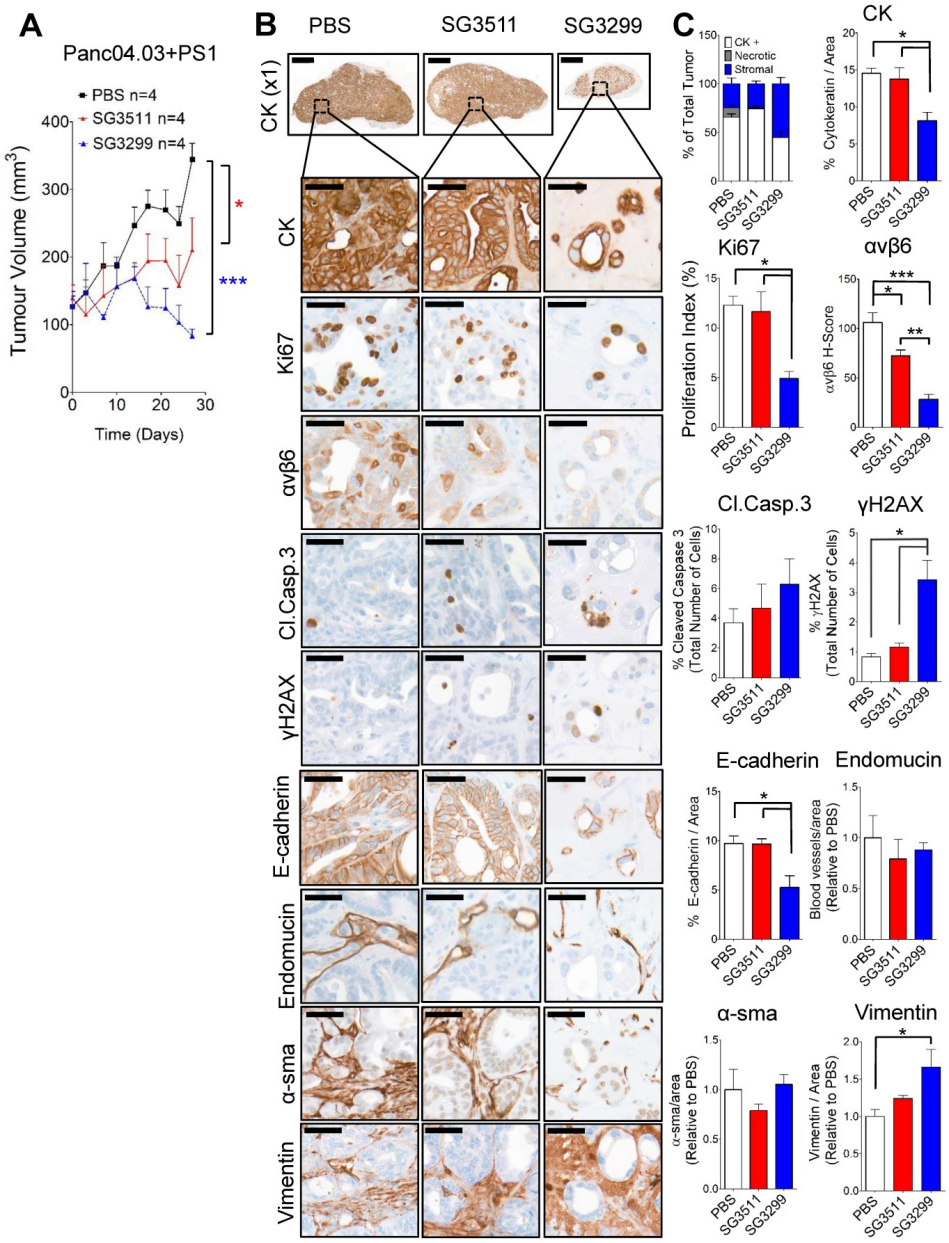
** Pancreatic stellate cells do not prevent the anti-tumorigenic action of SG3299. A,** Athymic CD1Nu/Nu female mice bearing human Panc0403+PS1 xenograft tumours were treated with NT-peptide (black square), SG3511 (red filled triangle) or SG3299 (blue unfilled triangle) (20 μg/kg; i.p administration) bi-weekly for 4 consecutive weeks (start of treatment day 0). Data are presented as mean tumor volume (error bars represent SEM, n=4 mice/group). Treatment commenced when tumors reached 100 mm^3^. **B,** Micrographs of Panc0403+PS1 tumor xenografts from mice treated as in (A) (n=4/treatment, after 4 weeks treatment). Tumors were harvested, fixed, paraffin-embedded and sections subject to immunohistochemical staining for the indicated molecules of interest including pan-cytokeratin (CK, epithelial marker), Ki67 (proliferation), αvβ6, cleaved-caspase 3 (Cl.Caspase 3; apoptosis), E-cadherin (epithelial marker), blood vasculature (endomucin), activated fibroblasts (α-sma), vimentin (stroma) and γH2AX (DNA damage/adduct formation). Representative images are shown of the 4 tumors harvested for each treatment. Magnification bar=2000 μM for x1 magnification images, magnified images (x50) are of indicated region of interest (tumor cells), with a scale bar of 50μM. **C,** Bar graph of composition of xenografts (% CK+ cells=white bar, % necrotic area in gray and % stroma is in blue) from (A) and histograms of marker expression shown in (B). Staining assessed & scored by 2 individuals or using VisioPharm computational analysis software (n=4 individual tumors, error bars represent SEM). *P=0.05, **P=0.01, ***P<0.001 (relative to PBS or indicated treatment).

PDAC tumours are characterised as having a high fraction of stroma rich in pancreatic stellate cells and mouse models should reflect this pathology 26. To validate the efficacy of the PDC SG3299 in a model that more closely reflects the human disease, we treated mice bearing tumors formed of a second αvβ6-positive PDAC cell line model, Panc0403, co-injected with human pancreatic stellate cells PS1 in a ratio of Panc0403:PS1 of 1:2. SG3299 significantly reduced Panc0403/PS1 xenograft tumor growth by 75.8±6% (P<0.001) compared with PBS treatment and by 60.4±9.8% (P<0.05) compared with SG3511 therapy (Figure 5A). Notably, Panc0403/PS1 tumour growth rate remained suppressed by SG3299 therapy even after treatment had ceased (Supplementary Figure S5).

### SG3299 treatment induced DNA damage and caused reduced proliferation and apoptosis in Panc0403/PS1 pancreatic tumors

As per Capan-1 xenograft studies, SG3299 was the only treatment to dramatically change tumor composition. SG3299 significantly increased DNA damage (γH2AX) by 5.5-fold and 2.9-fold (P<0.05) compared with PBS and SG3511 respectively (Figure 5B & C). As a likely consequence of this, SG3299 significantly reduced proliferation (Ki67) by 60±13.1% (P<0.05) and 54.8±13.8% (P<0.05) compared with PBS and SG3511 respectively. SG3299 also significantly reduced epithelial cell number (as measured by CK and E-cadherin) compared with PBS and SG3511 (44.2±18.1% and 41.2±19.1% reductions respectively for CK; 46±11% and 32.1±34.6% reductions respectively for E-cadherin, P<0.05). β6 expression was also significantly reduced with SG3299 treatment by 73.4±11% (P<0.001) compared to PBS and 61.1±16.1% (P<0.01) compared to SG3511.

## Discussion

Patients with pancreatic cancer are in desperate need of therapies that are more effective than the non-specific cyctotoxic drugs currently available. Integrin αvβ6 represents an exciting biomarker and therapeutic target in pancreatic cancer, especially as it was found to be highly expressed in almost 100% of PDAC cases tested 12-14, including the paired metastases 12. In this study we wished to develop a peptide-drug conjugate to treat pancreatic cancer.

Peptides are attractive for use as anti-cancer therapies as they are small, easy to synthesise and modify, have good biocompatibility and are more easily able to penetrate tumors than antibodies 19. Peptides are also rapidly excreted 27, thereby removing rapidly from the body any unused drug conjugated to the peptide, limiting toxicity to normal tissues. Additionally, the costs of treating patients with antibodies can be prohibitive 28, 29 whereas PDCs can offer a more favourable financial option. The observation that FMDV infects cattle using bovine αvβ6 30 led us to successfully identify A20FMDV2, a 20mer peptide that retained the integrin-specificity of the whole virus and the functionality, since A20FMDV2 binding promotes αvβ6-dependent internalisation into cells 7, 15, 16. Our study shows that αvβ6-specific peptide-drug conjugate SG3299 is an effective molecular-specific therapy for three different αvβ6-expressing tumour models including two PDAC xenografts.

PDCs have already been tested clinically in other cancers. Advanced endometrial (ZoptEC; NCT01767155), metastatic hormone-resistant prostate (NCT01240629) and metastatic breast and lung cancer (GRABM-B; NCT01480583 and GRABM-L; NCT01497665) have all received PDC therapies. However, none of these trials targeted a cancer-expressed antigen. These trials targeted the Leutenizing Releasing Hormone (LHRH) receptor, GnRH-R or the low density lipoprotein receptor-related protein 1 (LRP-1). The Phase 3 endometrial and Phase 2 GRABM-B trials failed to discern any survival benefit between standard of care and the PDCs combined with standards of care and thus the trials were terminated. These data suggest the choice of target for PDCs, as for any anti-cancer therapy, is key in the efficacy of the PDCs and emphasises the potential value of αvβ6 as a cancer-specific target.

The choice of warhead of PDCs is also critical in their likely success. PBD toxins, unlike other forms of chemotherapy, covalently cross-link both DNA strands simultaneously 18. This DNA inter-strand cross-link effectively blocks the division of cancer cells without distorting the structure of the DNA helix, thus avoiding triggering the DNA repair process and results in a longer duration of the cytotoxic effect 31. The cytotoxic effect of SG3299 requires DNA damage and this was observed in two different models of PDAC. This correlated with increased apoptosis and the associated reduction in tumour size and tumour cell proliferation. In agreement with these observed effects of SG3299, the data showed that after only three therapeutic treatments the Capan-1 tumour was mostly stromal fibroblasts (Figure 4).

The biology of pancreatic adenocarcinoma has suggested there are key components that must be targeted for any therapy to be effective. Thus, some PDAC cancer cells have been reported to possess 'stem-like' properties, associated with tumor initiation, metastasis and therapeutic resistance 24-26. Over 95% of the CD133-positive 'tumour initiating' cells in the PDAC PDX models used here were found to be αvβ6-positive (Figure 2A), suggesting targeting the αvβ6-positive population also targets the 'tumour initiating' cells. Data show that growing three-dimensional 'spheres' from individual PDAC cells *in vitro* enriches for the 'stem-like' phenotype 25. As SG3299 significantly reduced sphere-formation from PDAC PDX cells the results suggest these 'stem-like' cells are susceptible to SG3299. These findings are supported by previous studies where an antibody-PBD drug conjugate successfully eradicated tumor-initiating cells 32. Moreover, it is well established that PDAC tumours are mostly desmoplastic, formed predominantly of pancreatic stellate cells and their secreted stromal protein rich in collagen 33. Other data suggest that the pancreatic stellate cells may also promote drug resistance in pancreatic cancer 34. Therefore, we treated tumors formed from Panc0403 co-injected with human pancreatic stellate cells to mimic the human disease. Results showed that the presence of stellate cells did not inhibit the cytotoxic effect of SG3299, which still showed a strong therapeutic effect (Figure 5). Thus both 'stem-like' PDAC cells and pancreatic stellate cells do not prevent αvβ6-specific SG3299 mediating a significant therapeutic effect.

A previous study showed good evidence that αvβ6 functioned as a tumor suppressor in a transgenic mouse model of PDAC 35. This is in contrast to data in breast, cervix, colon and lung 8-11 suggesting αvβ6 functions as a tumor promotor, reducing the survival of patients that have cancers over-expressing this integrin. By using αvβ6 as a therapeutic target to selectively kill all αvβ6-expressing tumor cells, this apparent contradiction becomes irrelevant. Our data show that SG3299 can achieve that goal.

A new radiotracer, fluorine-18 radiolabelled A20FMDV2, was tested in clinical trials for detection of αvβ6 as part of an idiopathic pulmonary fibrosis (IPF) trial (NCT02612051). As αvβ6 is expressed in a wide range of malignancies, including pancreatic cancer 7,12, F18-A20FMDV2 or other recently described αvβ6-targeting radiotracers including from Hausner et al 36 and the cystine-knot R01 variant from the Gambhir Laboratory (Clinical Trials No. NCT02683824), offer non-invasive means of stratifying patients who may be eligible to receive anti-αvβ6 therapy. Promisingly SG3299 anti-cancer therapy showed no gross adverse effects in mouse weight, mouse behaviour or histologically in three different tissues that we previously have reported expressed αvβ6 in mice (reference 7 and Supplementary Figure S4); these data confirm that SG3299, targeting αvβ6, functioned as a cancer-specific therapy 37. The data also showed that when the dose and dosing schedule are optimal, SG3299 can be curative and a similar optimisation is likely to be critical for successful translation to humans. SG3299 has the potential to be therapeutically effective in PDAC and provide the first molecular-specific treatment for pancreatic cancer.

We have successfully translated the biological behaviour of the foot-and-mouth-disease virus into an effective anti-cancer therapy for human pancreatic cancer, and hopefully other types of cancer. With our development of αvβ6-specific peptide-drug conjugates such as SG3299, and the development of human 38 and humanised (trial ID NCT01371305) αvβ6-blocking antibodies, it is clear that specific targeting αvβ6 in humans is now a practical possibility and should become a platform for development of improved therapies for the effective treatment of pancreatic and hopefully many other types of cancer.

## Supplementary Material

Supplementary methods and figures.

## Figures and Tables

**Table 1:** Peptide-drug conjugate cytotoxicity *in vitro*

		Drug/ Peptide drug conjugate(PDC) (IC_50_ nM)^1^			PDC αvβ6 Specificity Ratio^2^
Cell Line	αvβ6 status	SG3199	SG3511	SG3299	
A375Ppuro	Negative	0.09 ± 0.07	101.08± 98.63	30.63 ± 18.75	3.30
A375Pβ6	Positive	0.11 ± 0.08	80.68 ± 41.65	5.37 ± 5.23	15.02
Capan-1	Positive	0.09 ± 0.08	11.72 ± 10.65	4.19 ± 3.76	2.80
Colo357	Positive	0.06 ± 0.05	24.57 ± 6.69	2.24 ± 1.59	10.97
Panc0403	Positive	0.55 ± 0.43	45.47 ± 17.61	3.44 ± 3.29	13.22
Panc1	Negative	1.70 ± 0.49	57.42 ± 11.56	175.57 ± 115.73	0.33

^1^IC_50_ values for drug alone (SG3199), non-targeting PDC (SG3511) and αvβ6-targeting PDC (SG3299) for targeting isogenic matched cell lines A375P-Puro and A375P-β6 and a panel of pancreatic cell lines (±SD, n=3). Cells were treated for 72h and subject to an MTT assay. ^2^Ratio of mean IC_50_ value for SG3511/SG3299.

## Abbreviations

α-sma: alpha smooth muscle actin
CK: cytokeratin
DMSO: dimethyl sulfoxide
FMDV: foot-and-mouth-disease virus
PDAC: pancreatic ductal adenocarcinoma
PBS: phosphate buffered saline
PDC: peptide drug conjugates
SD: standard deviation
