# Supplementary material for: Integrin αvβ6-specific therapy for pancreatic cancer developed from foot-and-mouth-disease virus
Source: Theranostics. 2020 Feb 12;10(7):2930–42. doi: 10.7150/thno.38702 (PMC7053198; doi:10.7150/thno.38702)
Supplement: Supplementary file 1 — Supplementary methods and figures. [file thnov10p2930s1.pdf]

Supplementary Methods

Analytical liquid chromatography/mass spectrometry (LC/MS) conditions (for reaction monitoring and purity determination) follows: Positive mode electrospray MS was performed using a Shimadzu Nexera®/Prominence® LCMS-2020. Mobile phases used were solvent A (H_2O/0.1% formic acid) and solvent B (CH_3CN/0.1% formic acid). Gradient for routine 3-min run: Initial composition 5%B held over 25s, increased from 5% B to 100% B over 1 min 35 s. Composition was held for 50 s at 100% B, returned to 5% B in 5 s and held for 5s. Total duration of gradient run was 3 min. Gradient for 15 min run: Initial composition 5% B held over 1min, increased from 5% B to 100% B over 9 min. Composition was held for 2 min at 100% B, returned to 5% B in 10 s and held for 2 min 50 s. Total duration of the gradient run was 15 min. Flow rate was 0.8 mL/minute (for 3 min run) and 0.6 mL/minute (for 15 min run). Detection was at 254 nm. Columns: Waters Acquity UPLC® BEH Shield RP18 1.7µm 2.1x50mm at 50°C fitted with Waters Acquity UPLC® BEH Shield RP18 VanGuard Pre-column, 130A, 1.7µm, 2.1mmx5mm (routine 3-min run); and ACE Excel 2 C18-AR, 2 µm, 3.0x100mm fitted with the Waters Acquity Pre-column as before (15-min run).

The preparative HPLC conditions were as follows: Reverse-phase ultra-fast high-performance liquid chromatography (UFLC) was carried out on a Shimazdzu Prominence® machine using a Phenomenex® Gemini NX 5µm C18 column(at 50°C) (150 x 21.2 mm). Eluents used were solvents A and B as previously described. All UFLC experiments were performed with gradient conditions: Initial composition 13% B increased to 60% B over 15 min then increased to 100% B over 2 min. Composition was held for 1min at 100% B, returned to 13% B in 0.1 min and held for

1.9 min. Total duration of gradient run was 20min. Flow rate was 20mL/minute and detection was at 254 and 280nm.

SG3511 (NT-peptide-SG3249)

SG3249 (0.018 mg, 12 µmol, 1.2eq.) and NT-peptide (0.024 mg,10 µmol,1.0 eq.) were dissolved in acetonitrile and 25mmol aqueous ammonium bicarbonate solution (1:1.3 mL). The mixture was stirred at RT for 1.5h where the reaction was shown to be complete by LCMS. Reaction mixture was lyophilised and purified by preparative HPLC to give the product as a white foam (0.017g, 43%). Analytical Data: LCMS 4.74 min (ES+) *m/z* (relative intensity) 1297 ($[M+2]^{+\cdot}$/3, 15%); 973 ($[M+3]^{+\cdot}$/4,100); 779 ($[M+4]^{+\cdot}$/5,60); 649 ($[M+5]^{+\cdot}$/6,50). The SG3511 HPLC peak (Supplementary Figure 1B) was 92.5% pure. Calculated molecular weight is 3990.06 g/mol.

SG3299 (A20FMDV2-SG3249)

SG3249 (0.0184 mg, 12.3 µmol, 1.2 eq.) and A20FMDV2 (0.0255 mg, 10.2µmol, 1.0 eq.) were processed as for SG3511 to give a white foam product (0.022 g, 54%). Analytical Data: LCMS 5.29 min (ES+) *m/z* (relative intensity) 1330 ($[M+2]^{+\cdot}$/3, 25%); 998 ($[M+3]^{+\cdot}$/4, 100); 798($[M+4]^{+\cdot}$/5,60); 665 ($[M+5]^{+\cdot}$/6,30). The SG3299 HPLC peak (Supplementary Figure 1B) was 93.1% pure. Calculated molecular weight is 3890.49 g/mol.

MTT cell cytotoxicity assay

Relative amounts of mitochondrial activity were quantified as an indirect measure of cell proliferation using the MTT assay (Promega). All cell lines were

seeded at $3x10^3$ cells/mL into 96-well plates. After 24h, cells were treated with serially-diluted concentrations of NT-peptide control, A20FMDV2, SG3199, SG3511 or SG3299 (0-500 nM, n=6 replicates/treatment) in serum-free media. Three biological repeats were performed. After 72 h, cells were subject to the MTT assay. Briefly, cell culture media was removed from each well and 100 µl of MTT reagent was added per well. After 1h at 37°C, MTT solution was replaced with 200µl DMSO and fluorescence measured at 550nm wavelength. Data were plotted relative to PBS (n=6, error bars represent standard deviation).

Flow cytometry

50 µl of $4x10^6$ cells/mL cell suspension in DMEM+0.1%BSA was incubated with 10D5 (Millipore), CD133 (Miltenyl) or mouse IgG (Millipore for 10D5, Biolegend for CD133) for 30 min, 4°C. Cells were washed twice in DMEM+0.1%BSA and then incubated with goat anti-mouse IgG conjugated to Alexa488 (Millipore) for 30 min in the dark at 4°C. Cells were washed and resuspended in DMEM+0.1%BSA and analysed for αvβ6 expression on a FACScalibur cytometer (Beckton-Dickinson). To measure binding of conjugates to cells, peptide-drugs were incubated with cells for 30min at 4°C. After washing off unbound material mouse anti-biotin antibody (200-002-211, Jackson ImmunoResearch) was added for 30 min before detecting with goat anti-mouse Alexa488 as above. Mean fluorescence intensity (MFI) was used as a measure of peptide binding in the isogenic matched cell lines.

Internalisation assay - Flow cytometry

$4x10^6$ cells/ml cells were seeded in serum-free DMEM. Cells were kept on ice and treated with 100 nM peptide-conjugate. After 15min cells were washed twice with DMEM+0.1%BSA prior to 15 min incubation with mouse anti-biotin (Jackson ImmunoResearch), then washed again. Apart from 0 time point, 30 min time point samples were incubated at 37°C, fixed in 4% formaldehyde/PBS for 10min, and then washed once in PBS. Samples were incubated for 15 min with goat anti-mouse IgG conjugated to Alexa488, in the dark at 4°C. Samples were washed as above and resuspended prior to analysis on a FACScalibur cytometer (Beckton-Dickinson). Reduction in fluorescence signal measured over time was associated with internalization of bound peptide as confirmed visually using confocal microscopy (see next subheading).

Internalisation assay – Immunocytochemistry & Confocal microscopy

$1x10^4$ cells/mL cells were seeded onto 13 mm coverslips and incubated at 37°C. After 24h cells were washed twice in ice cold serum-free DMEM. Cells were incubated on ice with 100 nM peptide in serum-free DMEM for 15 min, washed twice in serum-free DMEM prior to 15 min incubation with mouse anti-biotin. After two further washes, with the exception of the 0' time point, cells were fixed in 4% formaldehyde/PBS at 37°C for 10 min. Cells were then washed in PBS, followed by a 5 min incubation in 0.1%Triton X-100/PBS and a further PBS wash. All samples were then incubated in goat anti-mouse IgG-Alexa488, for 30 min in the dark. Samples were washed as before, followed by the addition of DAPI (40009, Biotium) and phallodin-TRITC (Sigma) for 10 min prior to mounting with Mowiol (81381, Sigma). Cells were visualised on the LSM710 Confocal Microscope (Zeiss) and images assessed for internalisation relative to the 0 min sample.

Immunohistochemical analysis quantification

Slides were scanned using a Pannoramic 250 Flask II high throughput scanner (3DHISTECH Ltd) and tumors assessed for their composition using the area selection tool and analyzer on the Pannoramic Viewer software (version 1.15.2, 3DHISTECH Ltd). Mean H-scores were counted manually for αvβ6 where tumors were graded according to area of staining (0-100%) multiplied by the intensity of that area (0=negative, 1=weak, 2=moderate, 3=strong), the value for each area of differential staining was then added together giving a final score range of 0-300. H-scoring was verified using DensitoQuant software in Pannoramic Viewer. Individual Ki67, cleaved-caspase 3 and γH2AX-positive tumor cells were counted using NuclearQuant software in Pannoramic Viewer.

All other markers were computationally scored using VisioPharm image analysis software (Visiopharm Integrator System, version 4.2.2.0, Visiopharm, Hoersholm, Denmark) using the company applications. For some markers (CK, E-cadherin, endomucin, α-sma and vimetin), the quantity of staining was normalized to the total live area of each tumor and further normalized to mean score for PBS tumors to give relative quantities compared to PBS treated tumors. Haematoxylin and eosin staining was also performed on lungs, intestine and stomach, which are subject to inflammation and hence express αvβ6, to observe any toxic effects on the gross morphology of these tissues.

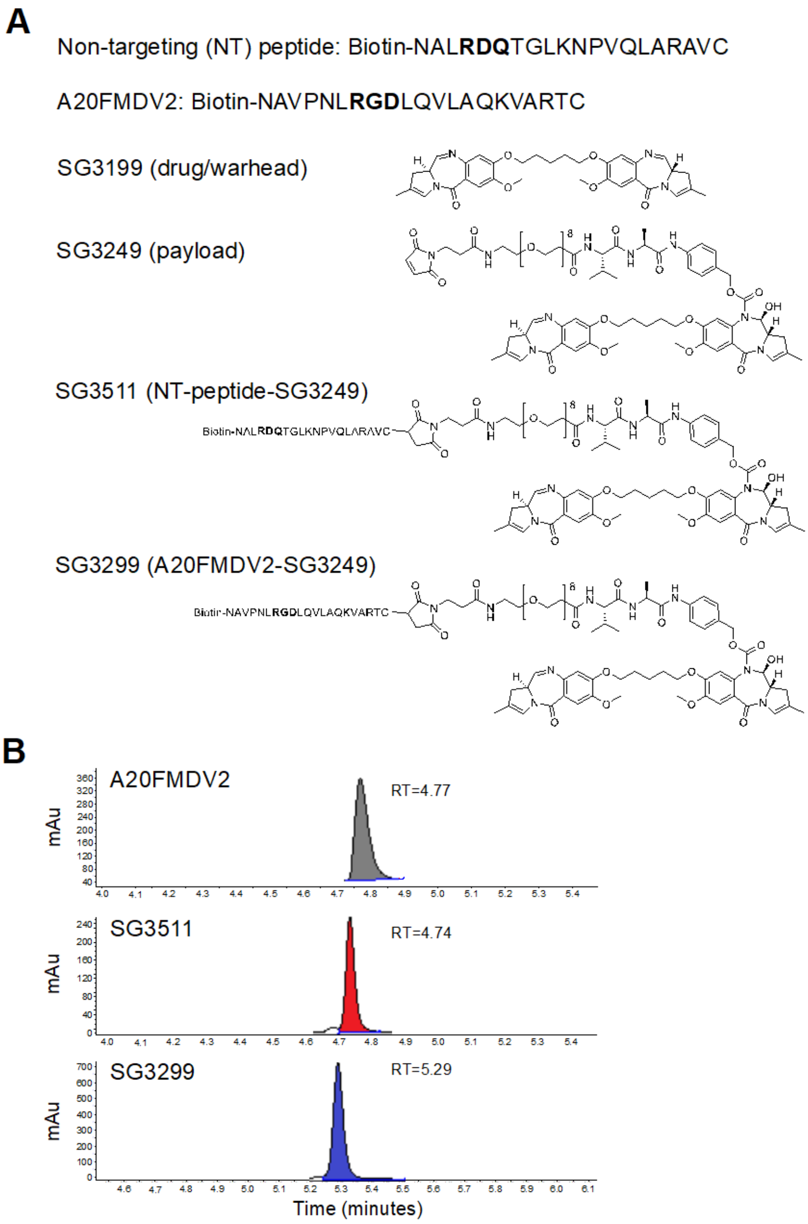

Supplementary Figure S1.Structure & Biochemistry of Peptide-Drug Conjugates.
A, Structural diagrams and amino acid sequences of non-targeting (NT) peptide, integrin αvβ6-specific peptide (A20FMDV2), warhead SG3199, payload SG3249, NT-peptide-SG3249 (SG3511) and A20FMDV2-SG3249 (SG3299). The position of the RGD αvβ6-specific binding site in A20FMDV2 and the altered sequence in NT-peptide are shown in bold. **B**, High-performance liquid chromatography confirmation of purity of A20FMDV2, SG3511 and SG3299, where RT is the retention time.

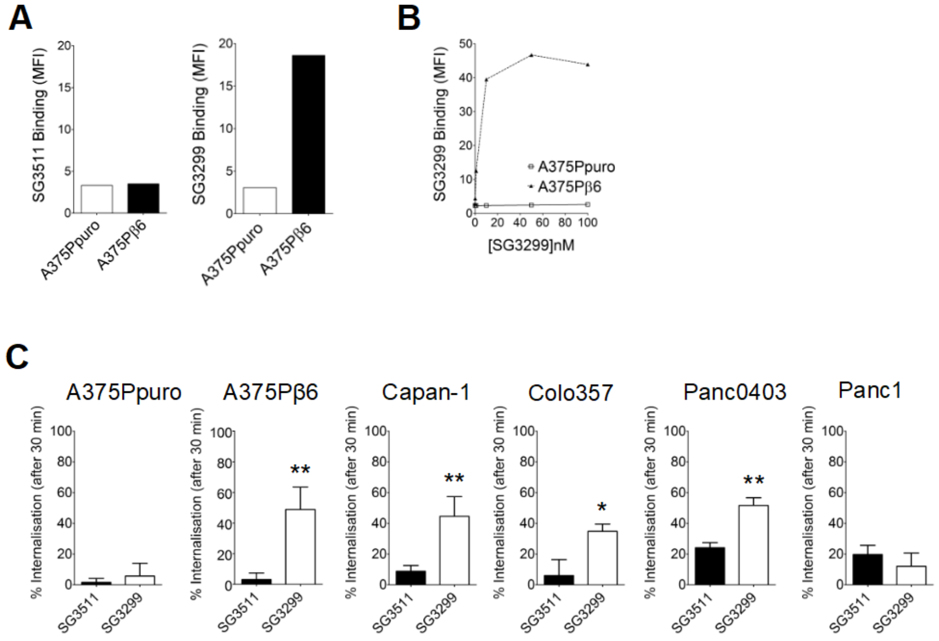

Supplementary Figure S2. Binding & Internalisation of Peptide-Conjugate is αvβ6-Specific. A, Binding of SG3511 and SG3299 at 100 nM and **B,** Dose-dependent binding of SG3299 in A375Ppuro and A375Pβ6 cells. **C,** Internalisation of SG3511 and SG3299 after 30 min in isogenic matched cell lines A375Ppuro and A375Pβ6 and a panel of pancreatic cell lines. **P*=0.05, ***P*=0.01 (n=3, error bars represent standard deviation).

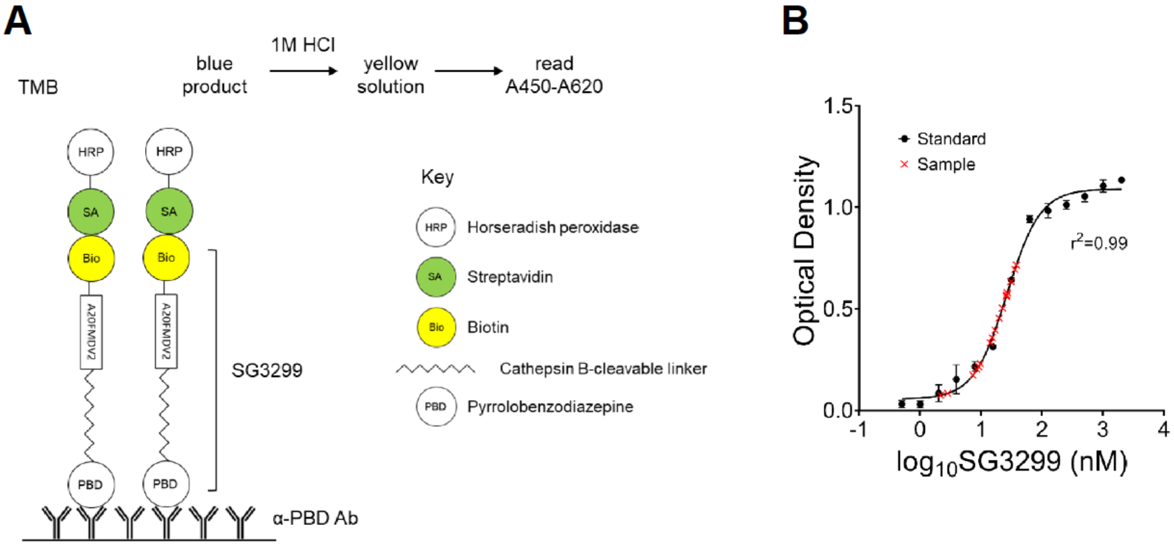

Supplementary Figure S3. Development of an ELISA for the detection of SG3299 from *in vivo* samples.

A, SG3299 ELISA design. Peptide-drug conjugate was captured via immobilised antibody raised against PBD and detected with Streptavidin-HRP via binding to the biotin tag. The addition of 3,3'5,5'-Tetramethylbenzidine (TMB) substrate followed by 1M HCl produced a yellow solution enabling the determination of SG3299 concentration by measurement of absorbance at 450nm minus absorbance at 620nm (defined as optical density). **B,** Standard curve for reconstituted SG3299 standards of known concentration and serum samples for data presented in Figure 3F.

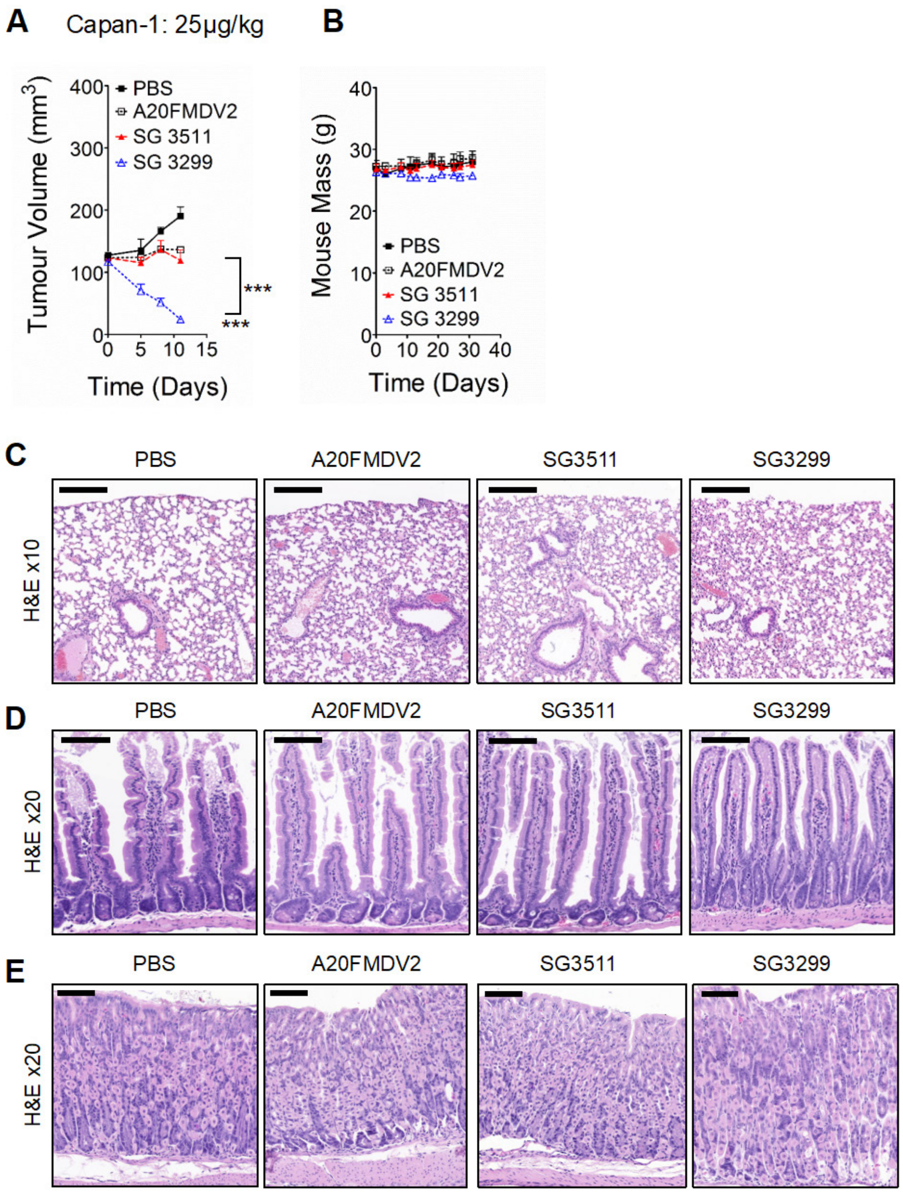

Supplementary Figure S4. SG3299 Therapy is Non-Toxic *In Vivo*.
A, Capan-1 cell line was grown as xenograft in 8 week old female CD1Nu/Nu mice at 2 x 10^6 cells/mouse (n=3/treatment, error bars are SEM). Once tumours reached ~100 mm^3 tumours were randomly allocated to a treatment group and were treated with PBS (black square), or 25 µg/kg A20FMDV2 (unfilled square), SG3511 (red triangle) or SG3299 (unfilled blue triangle) bi-weekly via i.p for 10 days (equating to three treatments) prior to harvesting for immunohistochemical analysis of tumor and other organs. **B**, Body weight of mice treated with peptide-drug conjugates as in (A) (mice from Figure 3D) over the course of 4 weeks treatment as a measure of animal welfare and peptide-drug conjugate toxicity (n=6-8/treatment, error bars are SEM). No adverse effects were noted in **C,** lung (bar=200µm), **D,** intestine or **E,** stomach (bar=100µm) (all αvβ6-positive tissues; n= 3 mice) assessed via H&E staining of tissues harvested from mice after receiving 25 µg/kg bi-weekly for 4 weeks (mice shown in Figure 3D).

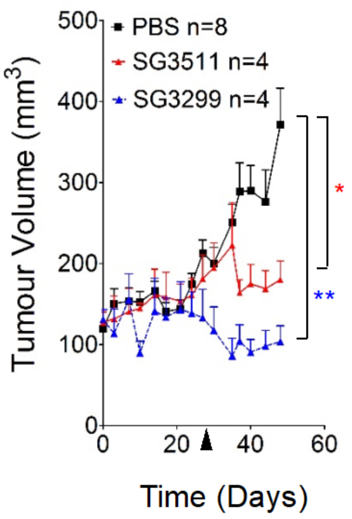

Supplementary Figure S5. SG3299 Selectively Kills αvβ6-Expressing Pancreatic Cancer Grown With Stellate Cells *In Vivo* Beyond Cessation Of Therapy.

Athymic CD1Nu/Nu female mice bearing human Panc0403+PS1 xenograft tumours were treated with NT-peptide (black square), SG3511 (red filled triangle) or SG3299 (blue unfilled triangle) (20 μg/kg; i.p administration) bi-weekly for 4 consecutive weeks (start of treatment day 0, arrow denotes when treatment ceased). Data are presented as mean tumor volume (error bars represent SEM, n=4-8 mice/group). Treatment commenced when tumors reached $100mm^3$. *P=0.05, **P=0.01 (n=3, error bars represent SEM).
